# “I Felt a Sense of Mission during Moments of Crisis”: Mental Health Professionals’ Perspectives on Their Initial Treatment of Evacuees during the Israel–Hamas Conflict

**DOI:** 10.3390/healthcare12111098

**Published:** 2024-05-27

**Authors:** Inbar Levkovich, Michal Labes

**Affiliations:** 1 Faculty of Graduate Studies, Oranim Academic College, Kiryat Tiv’on 36006, Israel; 2 Department of Criminology, Bar Ilan University, Ramat Gan 5290002, Israel; michal3m3@gmail.com

**Keywords:** mental health professionals, trauma, evacuations, qualitative study

## Abstract

This study examines mental health service providers who provided care to evacuees during the Israel–Hamas conflict. Utilizing a phenomenological qualitative method, the research delves into the psychological impact on the participants’ lived experiences. The sample included 25 mental health providers (13 female, age range 28–63, mean 42.4, SD 7.3; 15 psychologists and 10 social worker, average seniority 10.8 years, SD 5.2, range 2–18 years). Data were collected through semi-structured interviews conducted between December 2023 and March 2024. The data analysis revealed a dual narrative: Participants paid a major personal price and experienced secondary traumatization manifesting in emotional detachment, physical symptoms, and heightened arousal. They also derived a profound sense of meaning and fulfillment from their work, contributing to personal and professional growth. These findings underscore the complexity of their experiences, which were marked by the challenges of secondary trauma and the resilience fostered through their work. This study emphasizes the importance of support systems, including social and familial networks and professional supervision, in navigating these challenges. This study has several limitations, including small sample size and the use of virtual interviews, suggesting the need for further research with a broader participant base and in different contexts.

## 1. Introduction

Population evacuation is defined as the strategic relocation of individuals from high-risk areas owing to imminent threats to their safety, often caused by natural disasters or extreme security situations [1,2]. These individuals, referred to as evacuees, are forced to leave their homes in a hurry to seek refuge from impending crises [3]. This form of evacuation can have profound and enduring psychological consequences, with evacuees frequently recounting experiences fraught with distress and trauma [3,4,5,6]. Their traumatic exposure is correlated with a significant uptick in mental health disorders and post-traumatic stress disorder (PTSD) among evacuees, effectively doubling the rates observed within the general populace [7]. Hence, research studies have examined the experiences of these evacuees in an attempt to understand the psychological ramifications of population evacuation. The current study shifts the focus to the mental health professionals who were the first to treat these individuals. This study explores the emotional impact on the care providers themselves, including the challenges they face and their sources of support.

In recent decades, emergency evacuations, often prompted by natural disasters, have become common worldwide [8]. Notable examples include Hurricane Katrina in 2005, which severely affected New Orleans, Louisiana, resulting in over 1500 deaths, thousands of people displaced, and over 1.5 million individuals evacuated [1]. Similarly, in 2011, an earthquake and subsequent tsunami in Fukushima, Japan, destroyed coastal villages and forced approximately half a million people to be evacuated to makeshift facilities, many of whom did not return home [9]. On 7 October 2023, the conflict between Israel and Hamas intensified, causing damage to the populations on both sides and necessitating the evacuation of citizens from their places of residence [10,11,12]. The ensuing security debacle prompted the Israeli government to initiate an extensive evacuation protocol, relocating tens of thousands from affected zones to provisional shelters, thereby underscoring the immediacy and scale of such interventions [13].

In response to these events, mental health professionals swiftly mobilized to extend psychological support to evacuees, aimed at alleviating PTSD risks and fostering emotional stability [10]. Yet, these professionals were immediately confronted with content of a highly distressing nature, which posed a risk of secondary traumatization [14,15].

One primary concern that these professionals had to confront centered on the evacuees’ abrupt removal from their homes, which marked a fundamental shift that disrupted the notion of home as a symbol of stability and sanctuary. This kind of evacuation thrusts evacuees into uncertainty and precipitates profound emotional disturbances that detrimentally affect their interpersonal relationships, economic stability, familial bonds, and community connections [16,17]. This evacuation-induced sense of powerlessness is linked to elevated rates of anxiety, depression, and substance misuse among evacuees, relative to the general population [13,18].

Another critical concern for these mental health professionals was how to address the traumatic events that precipitated the need for psychological intervention and rehabilitation. Studies indicate that as many as 20% to 50% of evacuees may develop PTSD following exposure to trauma [7,13,19]. Research in the aftermath of 9/11 in the United States revealed that heightened exposure to traumatic events escalates the risk of PTSD, with factors such as low resilience and scant social support exacerbating this risk [20]. Similarly, after the October 7 attack in Israel, approximately 30% of evacuees exhibited symptoms indicative of PTSD [13]. Training professionals in disaster preparedness and response techniques before such incidents occur can foster resilience among work teams and improve the care provided to clients [21].

In such situations, the personal well-being of mental health professionals is also at stake, as continuous exposure to distressing narratives can manifest in physical symptoms such as headaches and hyperarousal, alongside emotional responses such as anger and anxiety [14,22]. Furthermore, there are similarities between the personal experiences of the mental health professionals and those of the evacuees, sharing a traumatic reality [23].

The security situation in the state directly influences mental health professionals who are dealing with stress, tension, anxiety, and fear for their own lives and those of their families [24]. Nevertheless, some mental health professionals may also experience elements of post-traumatic growth, such as an enhanced appreciation for life, a sense of professional efficacy, the establishment of new relationships, the discovery of personal strengths, and an enriched spiritual outlook [25,26].

The current study explored the impact of treating evacuees on mental health workers, from their own perspectives. It delved into the personal costs associated with their roles, the benefits they derived from providing care, and their sources of support. This study is innovative in its focus on the personal experiences of mental health professionals in assisting evacuees in the wake of disasters.

## 2. Materials and Methods

This qualitative study was conducted to explore the subjective experiences of mental health professionals who delivered therapeutic interventions to individuals displaced from their residences following the distressing events of the 7 October onslaught. This study employed a phenomenological approach to capture the essence and nuances of this phenomenon as experienced by those intimately involved. The analysis was grounded in the subjective perceptions and contextual realities of the participants, in line with Vagle’s (2018) conceptual framework of phenomenological methodologies [27].

### 2.1. Participants

From October 2023 to the present, the Israeli government has enacted a comprehensive evacuation protocol, relocating approximately 120,000 individuals from the north and south of the country to temporary shelters. Concurrently, mental health professionals were quickly mobilized to provide psychological support to those who were displaced. This study investigated the experiences of these professionals in addressing crises. The study employed purposive sampling to select participants who met the following inclusion criteria: certified mental health practitioners who held at least a bachelor’s degree in a relevant field and had experience assisting evacuees. The sample consisted of 25 mental health practitioners, with a gender distribution of 13 women and 12 men and an age range of 28 to 63 years (mean age = 42.4, SD = 7.3). Among the participants, 15 were psychologists, and 10 were social workers. Their average seniority was 10.8 years (SD = 5.2), with a range of 2 to 18 years. The participants lived in various residential areas: Northern Israel (n = 11, 44%), Central Israel (n = 8, 32%), and Southern Israel (n = 6, 24%). Three (12%) of these mental health professionals evacuated themselves, whereas the rest did not. The locations where assistance was provided to the evacuees were rural (n = 17, 68%) or urban (n = 9, 32%). Assistance was provided in non-governmental institutions such as associations and community centers (n = 19, 76%) or in state institutions such as hospitals (n = 6, 24%).

### 2.2. Data Collection

Data were collected through individual semi-structured interviews, a method that provided a thorough exploration of the participants’ lived experiences while allowing for adjustments in the flow of the interview as necessary [27]. The interviewers utilized a guide that served as a structural framework yet was sufficiently adaptable to foster natural dialogue and encourage meaningful self-expression between the interviewer and interviewee. The structured gradation of the research questions facilitated the building of trust, starting with general and informative queries and progressively moving to more personal topics. The interview questions were specifically designed to probe professional interactions and coping strategies when dealing with evacuees, drawing on both broad and targeted areas of inquiry (Table 1).

### 2.3. Research Procedure

After the institutional review board approved the study, participants were recruited via social media platforms and professional groups targeting Israeli mental health practitioners. Potential participants were briefed on the study’s goals and procedures and provided informed consent before participating. A doctoral student on the research team conducted the interviews via Zoom to ensure wide geographical representation. The interviews were conducted from December 2023 to March 2024. The interviews, conducted in Hebrew, lasted between 45 to 60 min and were transcribed verbatim for analysis. Quotations translated by a certified translator are presented in this article. Data collection continued until theoretical saturation was achieved, meaning that the subsequent interviews yielded no new material for analysis.

### 2.4. Data Analysis

The interview transcripts were subjected to thematic analysis [27] to identify and code the central themes and patterns. Two experienced qualitative researchers in the fields of psychology and education conducted the data analysis, one individual holding the rank of professor and the other in the process of completing a doctorate. The analysis was carried out in several stages:

A. Open coding: Each transcript was read line-by-line independently by the researchers, who noted the initial units of meaning (categories) emerging from the data. They assessed commonalities and differences across interviews and regrouped themes to represent major content areas that garnered significant attention from the participants.

B. Review and consensus: The researchers reviewed the identified larger themes, discussed any disparities, and sought consensus on theme content and interpretation.

C. Axial coding: During the second examination of the transcripts, researchers detected associations between themes and sub-themes related to context and content. They compared all the completed interviews to consolidate meaning and develop a theoretical construct.

D. Integration: In the final stage, the researchers conceptually reordered the main themes that emerged and reintroduced them within their relevant contexts.

This approach allowed for the examination of extensive data, facilitating the generation of generalizations and interpretations and the identification of the study’s central themes; the initial agreement rate between the two raters was high (approximately 89%). Disagreements were resolved through discussions between the co-authors and experts in the field.

## 3. Results

Three primary themes emerged from the qualitative examination of the interview data. The resulting themes and categories are summarized in Table 2. Additionally, Appendix A supplements the discussion with extra quotes from each theme.

### 3.1. Theme 1: “My Body Revealed I Was Struggling but I Was Unable to Stop”: The Personal Price Paid by Mental Health Professionals for Assisting Evacuees

The mental health professionals who assisted the evacuees reported that their work had a direct impact on their personal lives, exacted a significant personal toll, and even led to secondary traumatization. They described difficulties in daily functioning, including feelings of detachment and impatience toward their children and families. These professionals also experienced various physical symptoms, including heightened arousal, nausea, back and limb pain, sleep disturbances, and changes in appetite or instances of emotional eating.

*I recall waking up feeling terribly nauseated, as if all I wanted to do was vomit. I also experienced psychosomatic symptoms, including difficulty sleeping, headaches, and severe nausea*.

(Male psychologist, 31.)

*When I am home with my children, I tend to their needs and do everything that is expected of me. But is this always what I want to do? No. Even though I am physically present with them, am I genuinely available and fully engaged? Unfortunately, not as much as I would desire. I believe that if I worked in a different profession or under different circumstances, things might be different. On the surface, I appear to be functioning; I manage the household as if everything is in order—the house is clean, the laundry is done, and day-to-day operations run smoothly. Yet, the quality of my presence and engagement is not at the level I aspire to*.

(Female psychologist, 36.)

Mental health professionals reported that providing support to evacuees exposed them to distressing content that elicited feelings of fear and terror. They had no control over the content that emerged in their interactions with patients and were unavoidably exposed to harrowing narratives during therapy sessions. This exposure often resulted in detachment from their own emotions and sense of self. Many therapists expressed concerns that the profound distress they witnessed would lead to emotional numbness or paralysis. Consequently, they found solace in dedicating themselves to the well-being of the evacuees and channeled their efforts into the care they provided, albeit at the expense of their own emotional and physical well-being. This commitment offered them a sense of security, purpose, and empowerment. Yet, they also acknowledged that this approach led to self-neglect and they raised concerns about the potential deterioration of their own health and well-being.

*My total devotion to my work served as my defense mechanism. In situations like these, I felt compelled to stay busy; I couldn’t bring myself to stop. Even after the first week, when I fell seriously ill with a fever, I continued going to the hotel where the evacuees were housed. My work pace didn’t slow down; I just kept pushing through. I only felt the true impact of my illness during a friend’s wedding—that’s when I collapsed. It was then I realized the harm I was doing to myself, yet I felt trapped in a cycle of relentless action, as if I couldn’t do otherwise*.

(Female social worker, 29.)

It is important to note the differences between therapists with experience working with trauma and those without such experience. Analysis of the interviews revealed that therapists with prior experience in treating individuals who had undergone traumatic events demonstrated a better understanding of the emotional nuances that may arise during treatment. Consequently, they approached the treatment with greater resilience and preparedness. These therapists were adept at self-protection during sessions, established clear boundaries for their patients, and effectively supported their colleagues. In contrast, therapists lacking this experience described difficulties in regulating their emotions after hearing their patients’ stories, experiencing physical symptoms, and secondary traumatization.

*The very fact that I have had these experiences before with other patients—it sounds a little sad to say it, but I kind of know what I am getting into. I do not come with any illusion of rescue fantasies; I know the risk for me*.

(Female psychologist, 34.)

*I don’t have a background in trauma care. I’m a psychologist whose work is very diagnostic. So, when I volunteered, I felt insecure. In a war situation like this, I do not have the ability to separate my private life from that of my patients*.

(Male psychologist, 39.)

### 3.2. Theme 2: “Finding Meaning Amidst Disruption”: Benefits Derived from Assisting Evacuees

The mental health professionals who assisted the evacuees reported that despite the challenges and the exposure to distressing content, they found their work highly meaningful. Many reported that their support helped the evacuees cope with immediate challenges, reduced the risk of developing post-traumatic stress disorder, and promoted a sense of mental well-being. Being a source of support filled them with a sense of pride, a feeling of accomplishment, and a continued desire to help.

*I receive letters of thanks from families after they left the evacuation center or relocated. I keep these letters on my fridge; they are incredibly moving to me and the words touch me deeply. Considering the brief amount of time I knew these people, it’s astonishing how significant my support was to them. To think that a simple gesture of mine could serve as such a crucial anchor, that a brief embrace could mean so much, to the extent that they felt I was like family, is truly hard to believe*.

(Female psychotherapist, 47.)

*I was the sole care provider for children at that hotel. With some children, the approach involved play therapy, in which profound developments can occur even after only three or four sessions. This work imparts a sense of purpose, as if what we do truly has meaning and impact*.

(Female psychologist, 34.)

The mental health professionals stated that a key indicator of their success was their ability to engender trust in the therapeutic process among their patients. Initially, many evacuees were hesitant to engage, afraid to open up, and even resistant to the therapists’ interventions. Yet, as the therapy progressed, a shift occurred and the patients began placing their trust in the therapists. A substantial number of therapists acknowledged the challenge posed by the knowledge that their interactions with these patients would be brief. Therefore, they placed great emphasis on building trust in the therapeutic process, with the hope that it would encourage patients to pursue long-term treatment in the future.

*I was truly delighted and found it incredibly rewarding to learn that following our sessions the patient no longer experienced nightmares. But beyond that, I believe the most significant outcome was her newfound trust in the therapeutic process. She began to see that treatment is effective and valuable*.

(Male psychologist, 39.)

The mental health professionals reported that despite the challenging conditions, therapeutic successes were attained swiftly. Many conducted sessions in unconventional settings like hotel lobbies or other public spaces, where people other than just the therapist and patient might have been present. These conditions required the therapists to use mental agility, prompting them to learn, innovate, and refine their methods. Consequently, they gained a heightened sense of proficiency and confidence in their ability to aid individuals, reinforcing their faith in the capacity of the therapeutic process to empower evacuees.

*In some ways I feel that working in hotels required me to exercise much more mental flexibility, a kind of resilience so to speak, imbuing me with the feeling that yes, I am capable. I was there for a week and did things and helped people, I mean, it actually developed some kind of capability in me. This experience and this need really set me on fire, instilled me with vitality to take care of people*.

(Male psychologist, 31.)

### 3.3. Theme 3: “Self-Care Comes First”: Support Systems for Mental Health Professionals Assisting Evacuees

The mental health professionals emphasized that they relied on help from various support systems to deal with the complexities of treating evacuees. A key source of support mentioned by all mental health personnel was social support from friends and family. Some embarked on their mission to aid evacuees as part of a social group that served as a crucial support network and offered a space for venting, shared learning, and candid conversations. Others noted that returning to their families at the end of the workday provided a sense of normalcy, stability, and security. These social support systems played a vital role in sustaining the well-being of these professionals by helping mitigate feelings of isolation, sadness, and distress.

*I can honestly say that my saving grace was my close friends who were also volunteering. We would gather together in the evenings for much needed interactions. We formed singing circles in which we alternated between crying and laughing. That time was precious to us*.

(Female social worker, 29.)

The mental health professionals also noted that specialized supervision of their work with evacuee patients was a crucial support mechanism during this period. This supervision allowed them to consult with experts on treatment-related questions, share experiences, and strategize on continued patient care. Some of these professionals obtained this training through associations with which they were affiliated, while others invested in it privately. Those who lacked access to such training felt its absence keenly, recognizing its potential to aid in coping strategies.

Conversely, despite consulting regularly with other mental health professionals for their own well-being, many of the participating therapists found it challenging to maintain personal care while helping the evacuees. They feared becoming vulnerable at a time when they felt compelled to remain resilient, leading some to forego their own therapy sessions. This reluctance underscored a survival mindset among therapists, who believed it was imperative to conserve their strength to effectively address the immediate challenges.

*Supervision is essential for me. My weekly therapy sessions provided a space solely for me to sort through the chaos. But I must admit that at the onset of the conflict I paused my therapy sessions. It felt like therapy was too much to handle at that time... as if it could potentially unravel me more than it could rebuild me. I was still deeply engulfed in the situation and I felt it wasn’t the right time to delve into the deep-seated issues of pain, despair, loss, and grief. My nervous system was still in a heightened state of alert and emergency, like everyone else in the country*.

(Male social worker, 39.)

Another factor noted by the participating mental health professionals was the importance of maintaining a balanced lifestyle. Despite the chaos and intense workload, those who managed to obtain adequate sleep, maintain regular eating habits, engage in physical activity, and allocate time for hobbies reported that these practices made them more effective when working with evacuees. Conversely, those who neglected their basic needs experienced difficulties, exhibited symptoms of physical illness, and in some cases, engaged in self-neglect.

*Sometimes, listening to the evacuees’ stories was overwhelming. During this period I had very little spare energy. I felt as if all my capacities were being utilized with nothing left for other activities. Therefore, it was crucial for me to maintain daily practices like meditation, listening to music, going to bed early, eating well, and exercising occasionally*.

(Male social worker, 38.)

## 4. Discussion

This study aimed to explore the experiences of mental health practitioners who assisted Israeli evacuees displaced by the conflict between Hamas and Israel. The mental health professionals reported that their engagement with the evacuees significantly impacted their lives, often exacting a personal toll. They described experiencing complex and burdensome emotions, including stress, fear, restlessness, and heightened arousal, alongside physical symptoms like headaches, stomachaches, nausea, and sleep and appetite disturbances. These findings align with prior research on care providers who supported evacuees following disasters [15,28]. A qualitative study involving mental health professionals who aided 9/11 survivors revealed similar distress and emotional turmoil, with therapists experiencing stress, anger, and anxiety and often becoming visibly emotional when recounting their clients’ experiences [29].

Secondary traumatic stress (STS) is a possible explanation for these reactions. This refers to the trauma symptoms experienced by individuals who are indirectly exposed to traumatic events, often through hearing about the trauma or seeing related images and videos [15,28]. According to the DSM-5, symptoms of secondary traumatization are similar to those of post-traumatic stress and include both behavioral and emotional symptoms. Individuals experiencing secondary traumatization may suffer from intrusive thoughts about a traumatic event, high arousal, avoidance behaviors, and negative changes in thinking, perception of reality, and mood [29]. A meta-analysis showed that care providers near disaster sites exhibited symptoms of secondary traumatization, although these symptoms typically diminished over time. Despite this, 15–35% of those indirectly exposed to trauma developed symptoms of post-traumatic stress disorder (PTSD) [15]. In a study conducted among mental health therapists working during the war between Russia and Ukraine, it was found that approximately 50% of therapists experienced symptoms of secondary traumatization and high levels of anxiety [30].

Conversely, the current study also highlighted positive outcomes, including feelings of pride, success, and satisfaction among mental health professionals, who attributed these to their work with evacuees. They reported experiencing personal and spiritual growth, a renewed sense of mission, and a restoration of control that had been lost in the wake of the conflict. These positive effects resonate with previous literature findings, suggesting that therapists who work with disaster victims can experience personal and professional growth characterized by increased self-confidence, life appreciation, and enhanced professional skills [26,31,32].

The concept of vicarious post-traumatic growth (VPTG) provides a framework for understanding these positive changes. VPTG posits that individuals can experience psychological growth following exposure to the traumatic experiences of others. This growth involves cognitive and intentional re-evaluation of life narratives to adapt to the aftermath of trauma [26]. Thus, care providers can simultaneously exhibit symptoms of secondary trauma and post-traumatic growth [33]. Initially, therapists may experience stress and distress; however, over time, they often adopt a broader perspective that allows positive reinterpretation of their therapeutic encounters. Vicarious post-traumatic growth is predicated on the derivation of new meanings from encounters with pain and loss [26]. This growth can manifest across five dimensions: appreciation of life; recognition of new possibilities and altered priorities; increased mental strength; changes in relationships with others; and spiritual development [33]. The dimensions of life appreciation and mental strength were particularly prominent in the current study. Mental health workers reported that their experiences working with evacuees enhanced their gratitude for their own lives and families and instilled a sense of value and vitality in their professional roles.

Support systems played a crucial role in helping the mental health professionals navigate the challenges associated with treating the evacuees. The participating therapists emphasized the importance of social and familial support and of targeted training in enhancing their efficacy and coping mechanisms. This finding is supported by the literature emphasizing the value of training and community support in crisis management and stress reduction among care providers [34]. A qualitative study in the United States also highlighted the benefits of adequate training and peer collaboration among social workers who helped evacuees after the hurricanes of 2017–2018 [35].

### 4.1. Limitations of the Study

This study employed a phenomenological qualitative approach to explore the experiences of mental health professionals who assisted Israeli evacuees during the conflict with Hamas. Given the nature of qualitative research, this study’s small sample size limits the generalizability of its findings to a broader population. Furthermore, the current study did not explore cultural, gender, or geographic residential differences among the participants. These factors may have impacted the results, and it is recommended that future research broaden the scope to include a comprehensive investigation of these variables. To overcome this limitation, subsequent studies should aim to diversify the participant pool by including mental health professionals from various disciplines and with varying levels of expertise. Another limitation of this study is the potential for social desirability bias, which is prevalent in social sciences. It is possible that mental health professionals may have emphasized certain aspects of their personality or work due to the nature of the interview or research context. Another limitation was the use of Zoom for conducting interviews, a measure adopted to ensure the safety of participants and researchers who needed to remain within the reach of missile-protected areas and to minimize travel during the conflict. While this approach was practical under these circumstances, it may have affected the depth and quality of the data collected compared with data from in-person interviews. We recommend exploring various data collection methods, such as focus groups, and broadening the scope to include perspectives of family members of mental health professionals who assisted evacuees. This approach would aim to enrich the resultant insights. Additionally, we advocate for conducting further studies in this field at different times and on a larger scale to enhance and build upon the findings of this study.

### 4.2. Conclusions

The study delves into the experiences of mental health professionals who supported evacuees during the conflict with Hamas. It highlights the significant personal and professional impact on these care providers, revealing a dual narrative of challenge and growth. On the one hand, these professionals paid substantial personal prices, including secondary traumatization and various physical and emotional symptoms, underscoring the intense burden of their work. On the other hand, this study also reveals a profound sense of fulfillment and positive psychological growth among these professionals that they attributed to their meaningful work with evacuees. The research emphasizes the complexity of their experiences, shaped by both the hardships encountered and the resilience developed through their pivotal roles in crisis intervention.

## Figures and Tables

**Table 1:** Interview questions posed to the participants.

How did you begin working with the evacuees?
How long have you been working with them?
Through what framework did you start working with the evacuees?
What were your living conditions during this time?
Were you compensated for your work with the evacuees?
What were the primary challenges you faced in your work with the evacuees?
Can you describe a significant event or story from this period?
Share a story or event that was particularly challenging for you.
What professional growth or changes did you experience during this time?
What were your predominant feelings throughout this period?
Describe a moment of personal crisis you experienced while working with the evacuees.
What reactions did you receive from your family or friends about your work with the evacuees? Did these reactions change over time?
What impact have the stories and events you were exposed to had on your personal life?
What personal journey have you undergone through your work with the evacuees?
What personal sacrifices did you feel you made because of your work with the evacuees?
What sources of support were crucial for you while working with the evacuees?
What strengths did you discover in yourself through this work?
What lessons from your work with the evacuees would you like to apply in your future professional endeavors?

**Table 2:** Summary of qualitative findings.

Theme	Categories
**Theme 1**	Challenges in daily activities post-evacuee treatment. Physical symptoms and stiffness from evacuee care. Emotional detachment during evacuee assistance. Protective strategies.
**Theme 2**	Professional development and skill acquisition. Psychological growth and enhanced sense of competence and purpose. Empowering evacuees to perceive therapy as beneficial and impactful.
**Theme 3**	Family and social support as key assistance. Patient-focused training for care providers. Peer emotional and social support.

## Data Availability

The datasets generated and analyzed during the current study are not publicly available due to privacy concerns but are available from the corresponding author on reasonable request.

## Appendix A

**Table A1:** Additional citations per theme.

Theme	Sample Citations
**Theme 1**	I worked with a group of children, including a four-year-old girl who refused to return her doll at the end of an activity. All the other children complied, but she did not. I asked for a moment alone with her and empathized with her difficulty in parting with the doll. Noticing my black shirt, she commented, “You know it’s not great to wear black.” Curious, I asked why. She solemnly replied, “If they shoot you in the head, then you can’t see the blood flowing, and they can’t help you.” After she left, I asked the kindergarten teacher for a few minutes alone and I cried intensely. (Female psychologist, 63.) From a certain point, I found it overwhelming. Committing to this therapeutic process became increasingly difficult for me. I yearned to retreat and disconnect from the emotional weight and pain of the stories. I noticed I was neglecting my own well-being, indicative of the heavy toll these narratives were taking. (Male psychologist, 37.) The emotional toll is immense. Each story is extreme and blurs into the next, making it challenging to detach. I find myself constantly affected by these narratives. (Female social worker, 44.)
**Theme 2**	I once had to intervene with two evacuee boys who were refusing to leave their hotel room. I did this with the consent and willingness of their parents, but it was a profound experience for me. Witnessing their harsh living conditions left me utterly shocked. It was a stark realization to see an entire family displaced from their home, confined to just one room. This experience had a profound impact on me; when I returned home, I felt an overwhelming sense of gratitude for my own circumstances. I felt protected and as if something was sheltering me. (Female psychologist, 34.) For me, this work feels like a mission, and I’m afraid that if I were to break down, I might not be able to put myself back together and continue. Currently, I am setting aside difficult stories and focusing on the people and actions at hand. I choose to see the joy and the positive outcomes that emerge from our interactions. (Male social worker, 41.)
**Theme 3**	As an employee, my frustration stems from the apparent lack of structure in managing our work. There seems to be an absence of formal training, and when questions arise, satisfactory answers are hard to come by. The workload is intense and undoubtedly necessitates additional support, yet it feels as though no one is genuinely invested in addressing these issues. (Female psychologist, 36.) Working with evacuees has made me realize the importance of bolstering my own resilience more than ever before. Despite my extensive experience in therapy over the years, I’ve never felt such an acute need for personal resilience. It has reached a point where not only do I recognize the necessity for peer support, but I also understand the critical importance of self-care, especially when faced with the challenging narratives I encounter daily. (Female psychologist, 42.)
